# Scintillation proximity assay (SPA) as a new approach to determine a ligand’s kinetic profile. A case in point for the adenosine A_1_ receptor

**DOI:** 10.1007/s11302-015-9485-0

**Published:** 2015-12-09

**Authors:** Lizi Xia, Henk de Vries, Ad P. IJzerman, Laura H. Heitman

**Affiliations:** Division of Medicinal Chemistry, Leiden Academic Centre for Drug Research, Leiden University, P.O. Box 9502, 2300 RA Leiden, The Netherlands

**Keywords:** G protein-coupled receptors, Scintillation proximity assay, Binding kinetics, Adenosine A_1_ receptor, Residence time

## Abstract

Scintillation proximity assay (SPA) is a radio-isotopic technology format used to measure a wide range of biological interactions, including drug-target binding affinity studies. The assay is homogeneous in nature, as it relies on a “mix and measure” format. It does not involve a filtration step to separate bound from free ligand as is the case in a traditional receptor-binding assay. For G protein-coupled receptors (GPCRs), it has been shown that optimal binding kinetics, next to a high affinity of a ligand, can result in more desirable pharmacological profiles. However, traditional techniques to assess kinetic parameters tend to be cumbersome and laborious. We thus aimed to evaluate whether SPA can be an alternative platform for real-time receptor-binding kinetic measurements on GPCRs. To do so, we first validated the SPA technology for equilibrium binding studies on a prototypic class A GPCR, the human adenosine A_1_ receptor (hA_1_R). Differently to classic kinetic studies, the SPA technology allowed us to study binding kinetic processes almost real time, which is impossible in the filtration assay. To demonstrate the reliability of this technology for kinetic purposes, we performed the so-called competition association experiments. The association and dissociation rate constants (*k*_on_ and *k*_off_) of unlabeled hA_1_R ligands were reliably and quickly determined and agreed very well with the same parameters from a traditional filtration assay performed simultaneously. In conclusion, SPA is a very promising technique to determine the kinetic profile of the drug-target interaction. Its robustness and potential for high-throughput may render this technology a preferred choice for further kinetic studies.

## Introduction

Scintillation proximity assays (SPA) are a bead-based assay technology for radioligand binding studies in drug research [1, 2]. The technology is homogeneous in nature, as it relies on a “mix and measure” format and avoids a filtration step to separate bound from unbound radiolabeled ligand as is the case in a traditional receptor-binding assay [3]. SPA technology, therefore, allows the rapid and convenient assay of a wide range of molecular interactions in a homogeneous system [4, 5]. With the help of a suitable radiolabeled probe, the affinity of a compound for its drug target, such as G protein-coupled receptors (GPCRs), can be determined fast and reliably by SPA technology [6–9]. Nowadays in industry, SPA technology is routinely used for radioligand binding assays to determine ligand affinity in drug screening applications where high-throughput is required [10].

Alongside classical affinity parameters such as IC_50_ and *K*_i_ values, drug-target binding kinetics, in particular, the receptor-ligand residence time (RT) is emerging as an additional parameter to assess the therapeutic potential of drug candidates with respect to drug efficacy and safety [11–14]. Consequently, there is an increasing awareness of the importance of measuring the kinetics of drug-target interactions. In the research field of GPCRs, a number of structure-kinetic relationship (SKR) studies have been published that suggest that for educated compound triage for further studies binding kinetics should be included in the decision process [15–17]. Therefore, a fast and trustful approach to determine kinetic parameters is urgently required.

By definition, the RT is inversely proportional to the ligand dissociation rate constant (*k*_off_). This rate constant together with the association-rate constant (*k*_on_) can both be retrieved from appropriate kinetic experiments following the principles laid out by Motulsky and Mahan [18]. In that publication, so-called competition association experiments are described, which are conventionally performed in the form of filtration assays. In this format, the method consumes a great amount of radioligand, membrane protein, and other materials. Besides, the tediousness and limited throughput of the kinetic assay are impediments to obtain *k*_on_ and *k*_off_ values for series of ligands efficiently.

So far, there have been quite a few attempts to improve the efficiency of kinetic screening. For example, an insurmountable effect of slowly dissociating ligands in a functional IP-1 assay in SPA format on the neurokinin-1 (NK_1_) receptor has been described [19]. In this case, the costly functional methodology only allowed for the qualitative screening of the slowly dissociating ligands. Another kinetic screening approach in the form of SPA technology took the observation of a *K*_i_ (leftward) shift [20] over time as proof for slowly dissociating compounds from different GPCRs [6, 7]. Although SPA technology was used in both studies, they were essentially equilibrium binding assays with long incubation times (10 h in gonadotropin-releasing hormone (GnRH) receptor [6] and 5 h in human CCR5 receptor [7]).

Recently, a method called dual-point competition association assay that enables the relatively fast kinetic screening of series of compounds was introduced by Guo et al. [21]. By measuring radioligand binding at two different time points in the absence or presence of unlabeled competitors, the kinetic rate index (KRI) was obtained. Although both fast and slowly dissociating ligands can be characterized and discriminated with this index, it is still a rather qualitative measure, as the *k*_on_ (*k*_3_) and *k*_off_ (*k*_4_) values of the unlabeled ligands cannot be obtained. Thus, the resolution of a kinetic comparison for SKR through KRI values is not as high as with full kinetic parameters [17].

The true benefit of SPA technology relies in its separation-free approach, which could allow almost continuous kinetic measurements over time. Previously, a kinetic study of radioligand association and dissociation by SPA technology has been reported for the inositol trisphosphate receptor (InsP_3_R), a Ca^2+^ channel, and it indeed confirmed that SPA is a useful technique to determine fast *k*_on_ and *k*_off_ values that might have been difficult to obtain using traditional methods [22]. However, SPA technology as a format to study the kinetics of radioligand binding to GPCRs has not been reported in any detail.

Therefore, in the present study, we aimed to fill this gap by converting a filtration-based kinetic radioligand binding assay to an SPA format, using a prototypical GPCR, the human adenosine A_1_ receptor (hA_1_R), as an example. We firstly validated the SPA technology for equilibrium binding studies by comparing it to traditional filtration assays performed simultaneously. In these experiments, both hA_1_R agonists and antagonists were tested, and their affinity determined with SPA technology was similar to the affinity determined in a filtration assay. In subsequent kinetic studies, the SPA technology was of great benefit, as it allowed us to follow radioligand binding over time in a single well, which is impossible in the filtration assay. We further demonstrated advantage in the most laborious of all kinetic assays, the competition association experiment. The association and dissociation rate constants of unlabeled ligands for hA_1_R were reliably and quickly determined and agreed very well with the same parameters in a traditional filtration assay performed in parallel.

## Materials and methods

### Chemicals and reagents

[^3^H]-1,3-Dipropyl-8-cyclopentyl-xanthine ([^3^H]-DPCPX, specific activity 113.4 Ci · mmol^−1^) was purchased from ARC, Inc. (St. Louis, MO). The Wheat Germ Agglutinin-Polyvinyl toluene (WGA-PVT) SPA beads (RPNQ0001) were purchased from PerkinElmer (Waltham, MA). Adenosine deaminase (ADA) was purchased from Boehringer Mannheim (Mannheim, Germany). 1,3-Dipropyl-8-cyclopentyl-xanthine (DPCPX, a selective hA_1_R antagonist [23]), 8-cyclopentyl-3-*N*-[3-((3-(4-fluorosulphonyl)benzoyl)-oxy)-propyl]-1-*N*-propyl-xanthine (FSCPX, an irreversible hA_1_R antagonist [24]), 2-chloro-*N*^6^-cyclopentyladenosine (CCPA, a selective hA_1_R agonist [25]), *N*^6^-cyclopentyladenosine (CPA, a selective hA_1_R agonist [25]), 5′-*N*-ethylcarboxamidoadenosine (NECA, a non-selective agonist for adenosine receptors [26]), and guanosine-5′-triphosphate (GTP) were purchased from Sigma (St. Louis, MO). BCA (bicinchoninic acid) protein assay kit was obtained from Pierce Chemical Company (Rockford, IL). LUF5834 (an hA_1_R partial agonist) was synthesized in our laboratory as described previously [27]. Chinese hamster ovary (CHO) cells stably expressing the hA_1_R were obtained from Prof. Steve Hill (University of Nottingham, UK). All other chemicals were of analytical grade and obtained from standard commercial sources.

### Cell culture and membrane preparation

CHO cells stably expressing hA_1_R were grown in Ham’s F12 medium containing 10 % (*v* · *v*^−1^) normal adult bovine serum, streptomycin (100 μg · mL^−1^), penicillin (100 IU · mL^−1^), and G418 (0.4 mg · mL^−1^) at 37 °C in 5 % CO_2_. Cells were subcultured twice weekly at a ratio of 1:20 on 10-cm ø culture plates. For membrane preparation, cells were subcultured 1:10 and then transferred to 15-cm ø plates. Cells grown to 80 to 90 % confluency were detached from plates by scraping them into 5 mL phosphate-buffered saline (PBS), collected, and centrifuged at 700 g (3 000 rpm) for 5 min. Cell pellets derived from 30 plates were pooled and resuspended in 20 mL of ice-cold 25 mM Tris-HCl buffer (pH 7.4). An UltraThurrax (Heidolph Instruments, Schwabach, Germany) was used to homogenize the cell suspension. Membranes and the cytosolic fraction were separated by centrifugation at 100,000*g* (31 000 rpm) in a Beckman Optima LE-80K ultracentrifuge (Beckman Coulter, Fullerton, CA) at 4 °C for 20 min. The pellet was resuspended in 15 mL of the Tris-HCl buffer, and the homogenization and centrifugation step was repeated. Tris-HCl buffer (10 mL, pH 7.4) was used to resuspend the pellet, and ADA was added (0.8 IU · mL^−1^) to break down endogenous adenosine. Membranes were stored in 250 μL aliquots at −80 °C. Concentrations of membrane protein were measured using the BCA method [28].

### Radioligand displacement experiments

The displacement experiments were performed using 10 concentrations of competing ligands in 25 μL of assay buffer (For antagonists: 50 mM Tris-HCl [pH 7.4 at 25 °C]; for agonists: 50 mM Tris-HCl supplemented with 5 mM MgCl_2_ [pH 7.4]) in the presence of another 25 μL of assay buffer with a final concentration of 2.4 nM [^3^H]-DPCPX. At this concentration, total radioligand binding did not exceed 10 % of that added to prevent ligand depletion. Non-specific binding (NSB) was determined in the presence of 100 μM CPA. Each condition was measured in duplicate, and at least three individual experiments were performed.

#### The SPA technology

A mixture of 5 μg protein membrane and 1 mg SPA bead was pre-coupled in a shaker (Vibrax VXR, IKA) in a volume of 50 μL of assay buffer at room temperature for 30 min. Then, together with the radioligand and competing ligands, the membrane-bead mixture was dispatched in an Isoplate-96 Microplate (Perkin Elmer, Groningen, the Netherlands), in a final reaction volume of 100 μL. The plate was incubated for 1 h inside the counting chamber of a 2450 MicroBeta^2^ Plate Counter (Perkin Elmer, Groningen, the Netherlands) at the ambient temperature of 28 °C. The binding values were recorded in corrected counts per minute (CCPM).

#### The filtration assay

Membrane aliquots containing 5 μg protein were incubated together with the radioligand and competing ligands in a total volume of 100 μL assay buffer in a 96-well plate. After 1 h incubation at room temperature, the incubation was terminated by rapid vacuum filtration to separate the bound and free radioligand through 96-well GF/B filter plates using a PerkinElmer Filtermate-harvester (Perkin Elmer, Groningen, the Netherlands). Filters were subsequently washed three times with ice-cold wash buffer (50 mM Tris-HCl [pH 7.4], supplemented with 5 mM MgCl_2_). After 30 min of dehydration of the filter plate at 50 °C, the filter-bound radioactivity was determined by scintillation spectrometry using the 2450 MicroBeta^2^ Plate Counter. The binding values were recorded in both counts per minute (CPM) and disintegrations per minute (DPM).

### Radioligand association and dissociation experiments

#### The SPA technology

The membrane-bead mixture was prepared as described under “Radioligand displacement experiments.” Once the membrane-bead mixture was added to the wells of an Isoplate-96 Microplate, measurements of radioligand bound to the receptor were started immediately and continued every 30 s for 1 h, using the 2450 MicroBeta^2^ Plate Counter. Subsequently, radioligand dissociation was initiated by the addition of 10 μM unlabeled CPA. Another 1 h of measurements at every 30 s was used to record the amount of radioligand still bound to the receptor. Samples were obtained as described under “Radioligand displacement experiments.”

#### The filtration assay

Association experiments were performed by incubating membrane aliquots containing 5 μg of protein in a total volume of 100 μL of assay buffer at 28 °C with 2.4 nM [^3^H]-DPCPX. The amount of radioligand bound to the receptor was measured at different time intervals during a total incubation of 1 h. Dissociation experiments were performed by preincubating membrane aliquots containing 5 μg of protein in a total volume of 100 μL of assay buffer for 1 h. After the preincubation, radioligand dissociation was initiated by the addition of 10 μM unlabeled CPA. The amount of radioligand still bound to the receptor was measured at various time intervals for a total of 1 h to ensure that full dissociation from hA_1_R was reached. Incubations were terminated, and samples were obtained as described under “Radioligand displacement experiments.”

### Competition association experiments

The binding kinetics of unlabeled ligands was quantified using the competition association assay based on the theoretical framework by Motulsky and Mahan [18]. In this experiment, one concentration of IC_50_ or three different concentrations of unlabeled competing ligands were tested—namely, at IC_25_, IC_50_, and IC_75_ determined from “Radioligand displacement experiments.” For (partial) agonists, 1 mM of GTP was present in the agonist assay buffer to ensure that agonist binding only occurred to the uncoupled form of hA_1_R [13]. The assay was performed by incubating in a total volume of 100 μL of assay buffer at 28 °C with 2.4 nM [^3^H]-DPCPX.

#### The SPA technology

The membrane-bead mixture was prepared as described under “Radioligand displacement experiments.” Once the membrane-bead mixture was added to the wells of an Isoplate-96 Microplate, measurements of radioligand bound to the receptor were started immediately and continued every 30 s for 2 h, using the 2450 MicroBeta^2^ Plate Counter. Samples were obtained as described under “Radioligand displacement experiments.”

#### The filtration assay

The competition association assay was initiated by adding membrane aliquots (5 μg per well) at different time points for a total of 2 h in the absence or presence of competing ligand. Incubations were terminated and samples were obtained as described under “Radioligand displacement experiments.”

### Data analysis

All values obtained are means of at least three independent experiments performed in duplicate. All experimental data were analyzed by using GraphPad Prism 6 (GraphPad Software, Inc., San Diego, CA), as in the description of previous work from our research group [21], including the following analysis: IC_50_ values obtained from competition displacement binding data were converted to *K*_i_ values using the Cheng-Prusoff equation [29], the *k*_on_ and *k*_off_ values for radiolabeled and unlabeled ligands were fitted and calculated, and the *k*_on_ and *k*_off_ values were used to calculate residence times (in min) and kinetic dissociation binding constants (kinetic *K*_D_).

## Results

### The affinity (*K*_i_) of hA_1_R ligands in displacement experiments

**Fig. 1:**
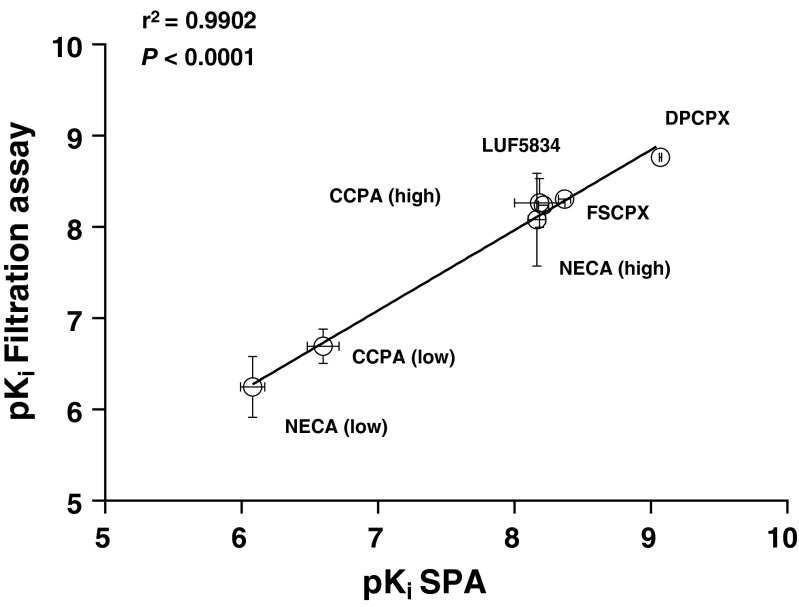
Correlation of the negative logarithm of hA_1_R ligands’ affinity (p*K*_i_) determined by SPA and in a filtration assay; agonists: CCPA, NECA, and LUF5834; antagonist: DPCPX and FSCPX

The affinities of several hA_1_R ligands were determined by displacement experiments formatted with SPA technology or as filtration assays. The tested hA_1_R ligands showed concentration-dependent inhibition of specific [^3^H]-DPCPX binding, and the data of antagonists (DPCPX, FSCPX) or partial agonist (LUF5834) were best fitted to a one-state competition model, while the data of full agonists (CCPA, NECA) were best fitted with a two-state receptor model. Affinities of all ligands determined by both SPA technology and filtration assay are shown in Table 1. All compounds showed high affinities, with those of antagonists and partial agonist in the nanomolar range. The agonists displayed high, nanomolar affinity for the so-called high affinity state, and lower, submicromolar affinity for the low affinity state. The affinities of the hA_1_R ligands from these equilibrium experiments were in good agreement between SPA technology and filtration assay (Fig. 1). Due to the irreversible binding characteristics of FSCPX, only its “apparent” affinity could be determined, which was subsequently included in the correlation.

**Table 1:** Comparison of the affinity of representative hA_1_R antagonists and (partial) agonists obtained from displacement studies of specific [^3^H]-DPCPX binding from hA_1_R membranes by SPA technology or filtration assay, respectively. Values are means ± s.e.m of at least three independent experiments performed in duplicate. For full agonists CCPA and NECA, displacement curves were best analyzed with a two-state model, yielding *K*_i_ values for a high affinity state and a low affinity state of the receptor

Compound	SPA *K*_i_ (nM)	Filtration *K*_i_ (nM)
FSCPX	0.9 ± 0.02^a^	1.6 ± 0.1^a^
DPCPX	4.3 ± 0.4	3.3 ± 0.3
LUF5834	6.2 ± 0.5	4.3 ± 0.6
CCPA	7.0 ± 1.1 (high)	8.3 ± 3.8 (high)
	861 ± 156 (low)	1010 ± 159 (low)
NECA	8.0 ± 2.3 (high)	7.8 ± 3.8 (high)
	282 ± 80 (low)	301 ± 39 (low)

^a^“Apparent” affinity of this irreversibly binding antagonist

### The association (*k*_on_) and dissociation rate constants (*k*_off_) of [^3^H]-DPCPX at hA_1_R

**Fig. 2:**
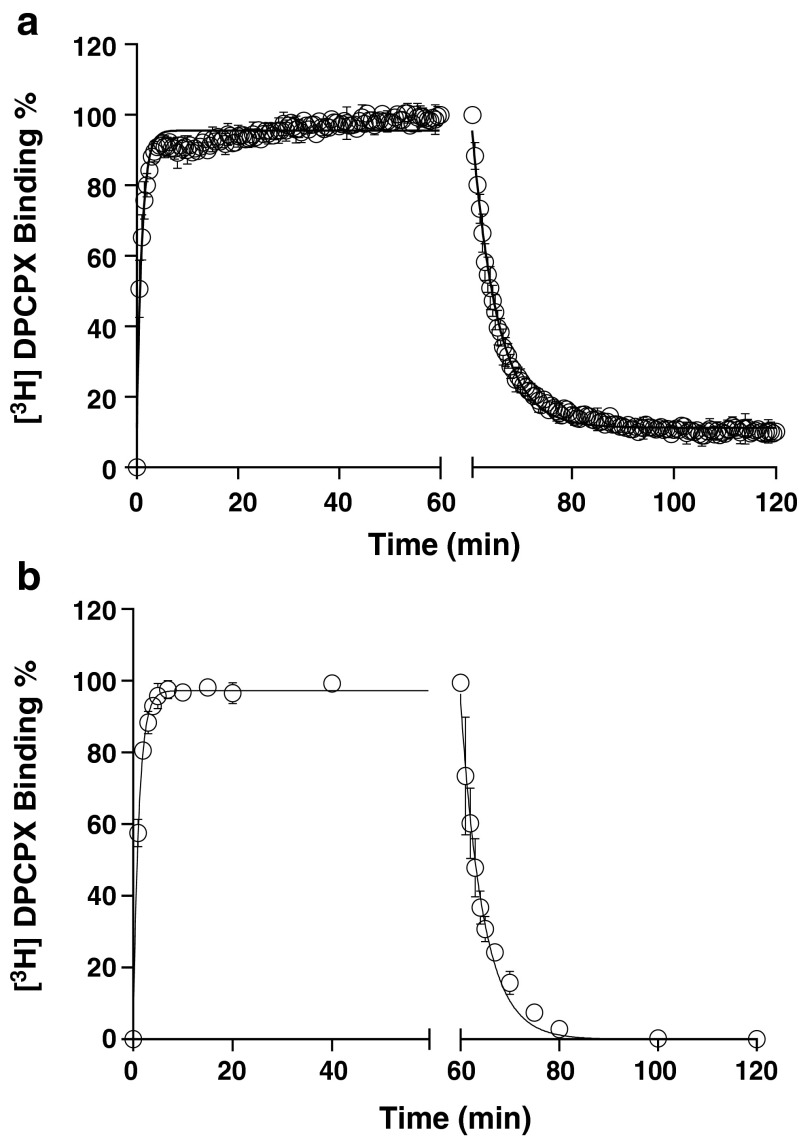
Association and dissociation kinetics of [^3^H]-DPCPX (2.4 nM) to and from hA_1_R stably expressed on CHO cell membranes (28 °C), measured in SPA technology (*n* = 3, combined and normalized, **a**) or filtration assay (*n* = 3, combined and normalized, **b**). 10 μM CPA was used as a displacer to initiate the dissociation. Association data was fitted in Prism 6 using one-phase exponential association. Dissociation data was fitted using one-phase exponential decay

Receptor association and dissociation rates of [^3^H]-DPCPX were directly determined in classic radioligand association and dissociation experiments with either SPA technology or filtration assays. In both assay formats, the binding of [^3^H]-DPCPX approached equilibrium after approximately 15 min (Fig. 2), indicating a relative fast *k*_on_ of 0.40 ± 0.05 nM^−1^ · min^−1^ by SPA technology and 0.24 ± 0.03 nM^−1^ · min^−1^ by filtration. Binding of the radioligand was reversible after the addition of 10 μM CPA, and complete dissociation was reached after approximately 25 min (Fig. 2). The *k*_off_ of [^3^H]-DPCPX from the hA_1_R was 0.20 ± 0.02 min^−1^ with SPA technology and 0.25 ± 0.01 min^−1^ in the filtration assay (Table 2). The kinetic *K*_D_ (*k*_off_/*k*_on_) of [^3^H]-DPCPX was 0.50 ± 0.08 nM (SPA) and 1.04 ± 0.14 nM (filtration) (Table 2). The residence time (RT, 1/*k*_off_) of [^3^H]-DPCPX was calculated as 5.0 ± 0.5 or 4.0 ± 0.2 min, determined by SPA or filtration, respectively.

**Table 2:** Comparison of the kinetic rates of [^3^H]-DPCPX obtained from classic kinetic association and dissociation experiments from hA_1_R membranes at 28 °C by SPA assay and filtration assay. Values are means ± s.e.m of three independent experiments performed in duplicate. Equations used are as follows: *k*_on_ = (*k*_obs_−*k*_off_)/[[^3^H]-DPCPX]; Kinetic *K*_D_ = *k*_off_/*k*_on_; RT = 1/*k*_off_. RT is residence time

[^3^H]-DPCPX	SPA	Filtration
*k*_on_ (nM^−1^ · min^−1^)	0.40 ± 0.05	0.24 ± 0.03
*k*_off_ (min^−1^)	0.20 ± 0.02	0.25 ± 0.01
Kinetic *K*_D_ (nM)	0.50 ± 0.08	1.0 ± 0.1
RT (min)	5.0 ± 0.5	4.0 ± 0.2

### The competition association assay at hA_1_R

**Fig. 3:**
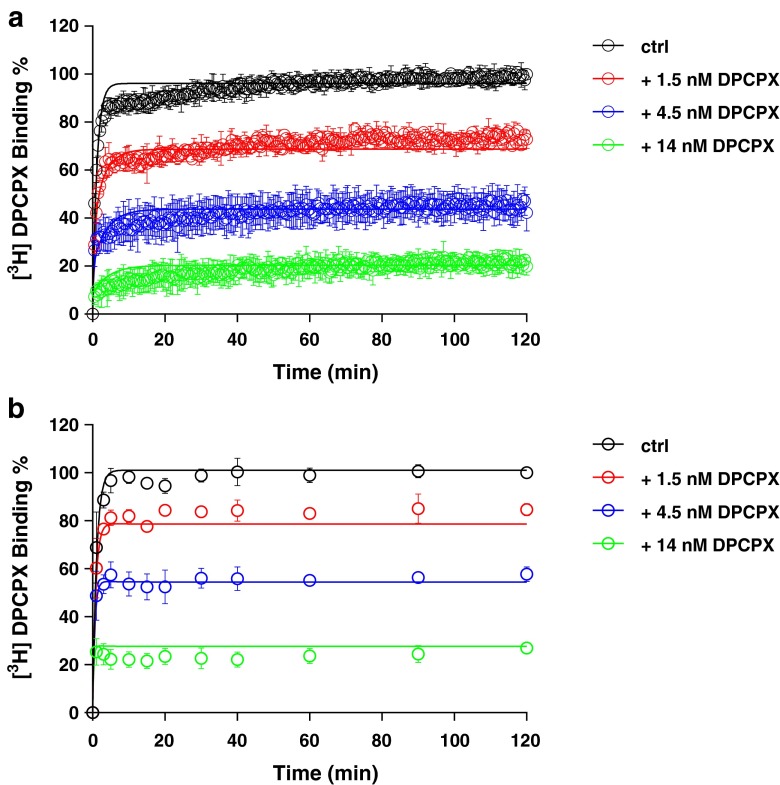
Competition association experiments with [^3^H]-DPCPX binding to hA_1_R stably expressed on CHO cell membranes (28 °C) in the absence or presence of 1.5, 4.5, and 14 nM of unlabeled DPCPX by SPA assay (*n* = 4, combined and normalized, **a**) or classic filtration assay (*n* = 3, combined and normalized, **b**)

With the established *k*_on_ (*k*_1_) and *k*_off_ (*k*_2_) values of [^3^H]-DPCPX binding from classic association and dissociation experiments, *k*_on_ (*k*_3_) and *k*_off_ (*k*_4_) values of unlabeled DPCPX were determined by fitting the values based on the mathematical model as previously described (see Materials and methods). Three different concentrations of unlabeled DPCPX, lower than (IC_25_), equal to and higher than (IC_75_) its IC_50_ value, were tested (Fig. 3). Its *k*_on_ and *k*_off_ values determined by this competition association method were 0.72 ± 0.16 nM^−1^ · min^−1^ and 0.50 ± 0.01 min^−1^ by SPA (Fig. 3a and Table 3) or 0.19 ± 0. 04 nM^−1^ · min^−1^ and 0.27 ± 0.03 min^−1^ by filtration (Fig. 3b and Table 3), which were in good accordance with the *k*_1_ and *k*_2_ values determined in the classic association and dissociation experiments (Table 2 and Fig. 2). Since the kinetic *K*_D_ values and affinities (*K*_i_) obtained from the different equilibrium and kinetic experiments are well comparable (Tables 1, 2, and 3), this further verified that the competition association assay by SPA technology could be accurately used to determine the binding kinetics of unlabeled A_1_R ligands.

**Table 3:** Comparison of the kinetic rate constants, residence times (RT) and kinetic *K*_D_ values of representative hA_1_R antagonists and (partial) agonists obtained from competition association experiments to hA_1_R expressed on CHO cell membranes at 28 °C by SPA assay and filtration assay. For (partial) agonists LUF5834, CCPA, and NECA, 1 mM GTP was present in the assay. The *k*_on_ (*k*_3_), *k*_off_ (*k*_4_) values of the unlabeled compounds were determined in [^3^H]-DPCPX (2.4 nM) competition association experiments. RTs and kinetic *K*_D_s were determined in the same manner as described in Table 2

Cmpd.	SPA				Filtration assay			
	*k*_on_ (nM^−1^ · min^−1^)	*k*_off_ (min^−1^)	RT (min)	Kinetic *K*_D_ (nM)	*k*_on_ (nM^−1^ · min^−1^)	*k*_off_ (min^−1^)	RT (min)	Kinetic *K*_D_ (nM)
FSCPX	0.0047 ± 0.0007	0.0064 ± 0.0013	156 ± 31	1.4 ± 0.3	0.0019 ± 0.0003	0.0060 ± 0.0020	167 ± 56	3.2 ± 0.4
DPCPX	0.72 ± 0.16	0.50 ± 0.01	2.0 ± 0.1	0.69 ± 0.15	0.19 ± 0. 04	0.27 ± 0.03	3.7 ± 0.4	1.4 ± 0.1
LUF5834	0.13 ± 0.05	0.50 ± 0.05	2.0 ± 0.1	3.9 ± 1.5	0.062 ± 0.006	0.23 ± 0.03	4.4 ± 0.5	3.7 ± 0.5
CCPA	0.0094 ± 0.0022	0.73 ± 0.04	1.4 ± 0.1	78 ± 19	0.016 ± 0.002	1.5 ± 0.03	0.68 ± 0.01	92 ± 9
NECA	0.0014 ± 0.0004	0.54 ± 0.06	1.9 ± 0.02	386 ± 36	0.0012 ± 0.0001	0.60 ± 0.04	1.7 ± 0.1	500 ± 8

**Fig. 4:**
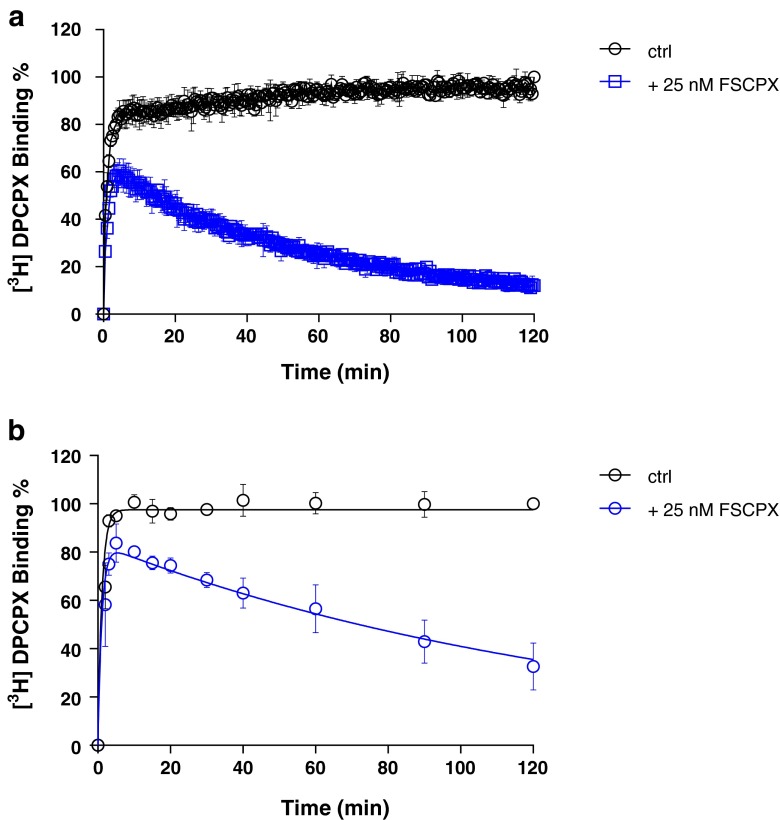
Competition association experiments with [^3^H]-DPCPX binding to hA_1_R stably expressed on CHO cell membranes (28 °C) in the absence or presence of 25 nM FSCPX, measured in SPA technology (*n* = 4, combined and normalized, **a**) or filtration assay (*n* = 3, combined and normalized, **b**)

We then used FSCPX, an irreversibly binding hA_1_R antagonist, as a further validation tool. In the competition association assay, FSCPX displayed an “overshoot” in the association curve indicating a negligible dissociation, which was observed in both SPA (Fig. 4a) and filtration assay (Fig. 4b). Its *k*_on_ and *k*_off_ values determined by the competition association method were 0.0047 ± 0.0007 nM^−1^ · min^−1^ and 0.0064 ± 0.0013 min^−1^ by SPA or 0.0019 ± 0.0003 nM^−1^ · min^−1^ and 0.0060 ± 0.0020 min^−1^ by filtration (Table 3).

**Fig. 5:**
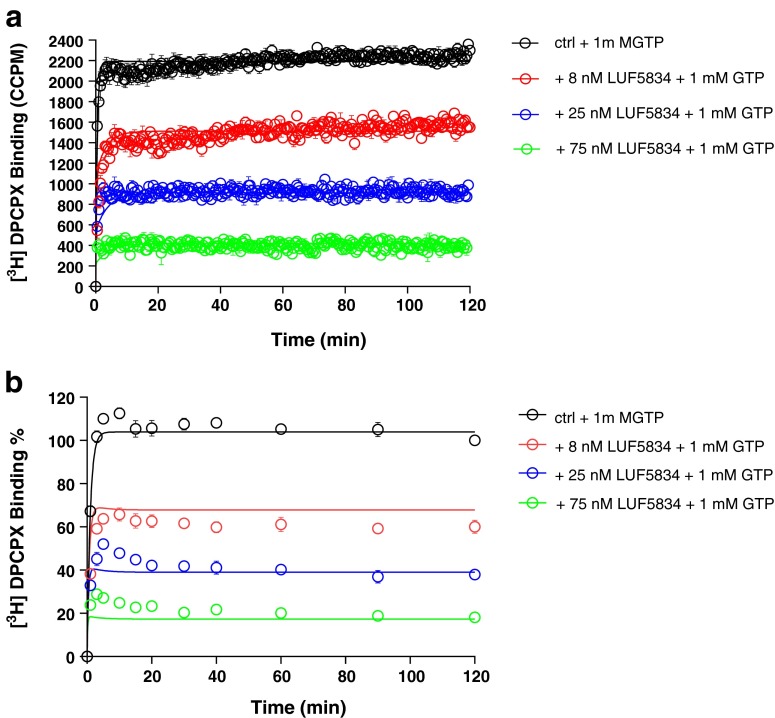
Competition association experiments with [^3^H]-DPCPX binding to hA_1_R stably expressed on CHO cell membranes (28 °C) in the absence or presence of 8, 25, and 75 nM of unlabeled LUF5834 by SPA technology (*n* = 3, one representative experiment, **a**) or filtration assay (n = 3, combined and normalized, **b**)

**Fig. 6:**
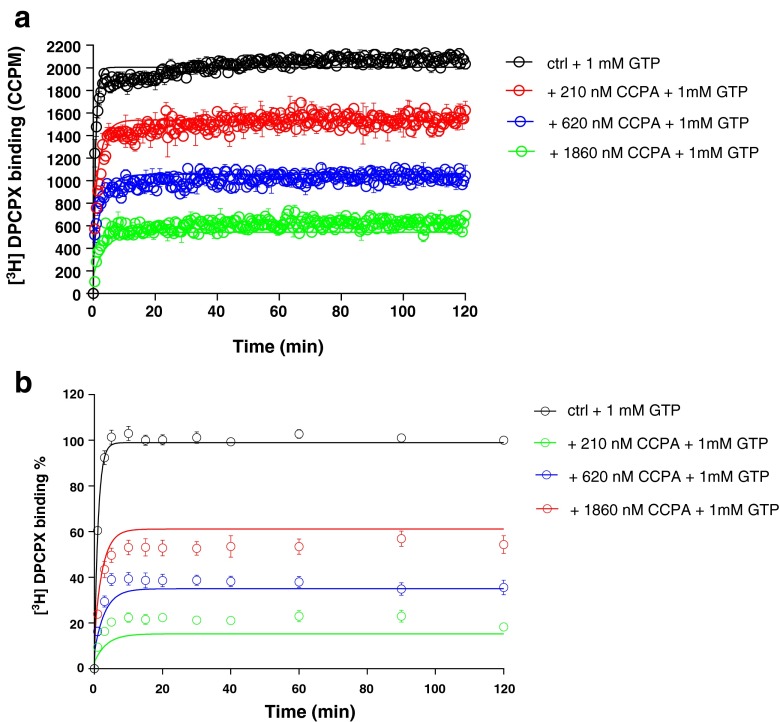
Competition association experiments with [^3^H]-DPCPX binding to hA_1_R stably expressed on CHO cell membranes (28 °C) in the absence or presence of 210, 620, and 1860 nM of unlabeled CCPA by SPA assay (*n* = 3, one representative experiment, **a**) or filtration assay (*n* = 3, combined and normalized, **b**)

**Fig. 7:**
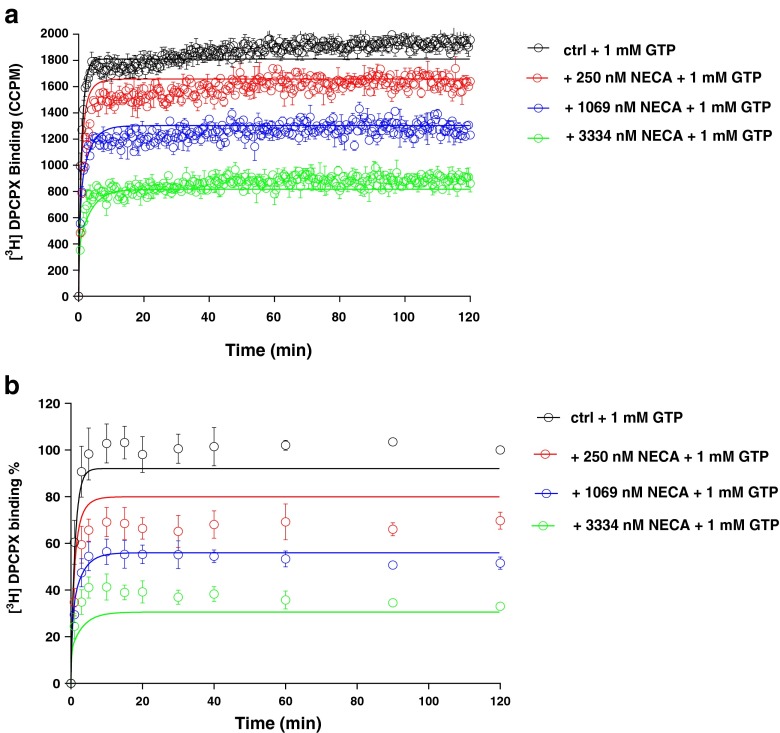
Competition association experiments with [^3^H]-DPCPX binding to hA_1_R stably expressed on CHO cell membranes (28 °C) in the absence or presence of 250, 1069, and 3334 nM of unlabeled NECA by SPA assay (*n* = 3, one representative experiment, **a**) or filtration assay (*n* = 3, combined and normalized, **b**)

**Fig. 8:**
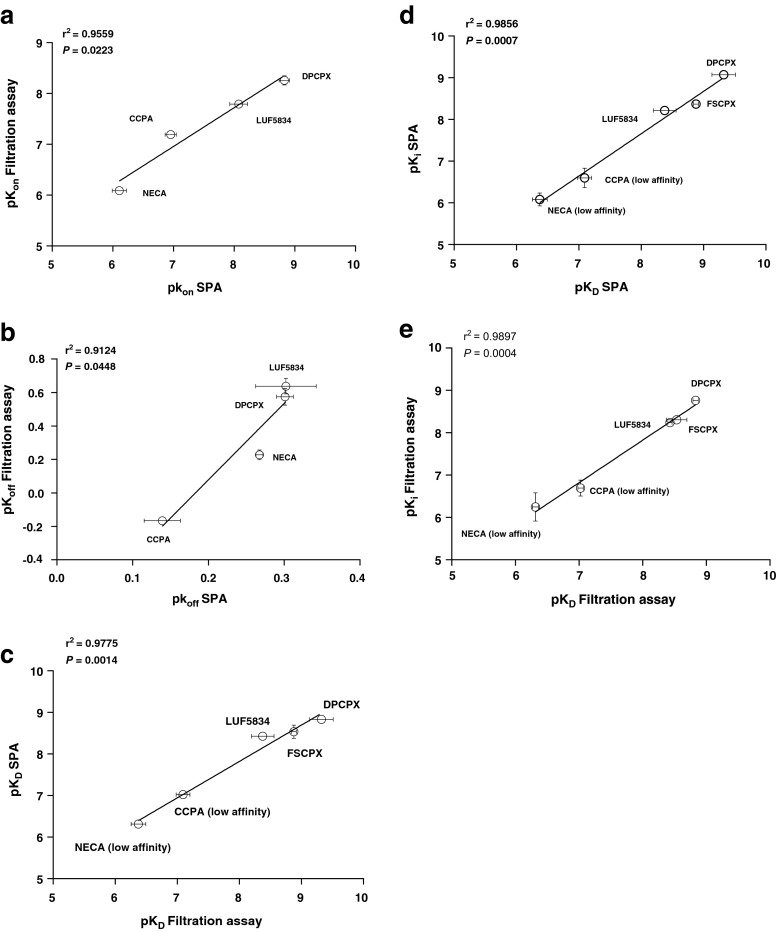
Correlation of the negative logarithm of hA_1_R ligands’ association rates (p*k*_on_, **a**) and dissociation rates (p*k*_off,_ **b**) determined by SPA (*x*-axis) and filtration assay (*y*-axis). Agonists: CCPA, NECA, and LUF5834; antagonist: DPCPX. Correlation of the negative logarithm of hA_1_R ligands’ kinetic *K*_D_ (p*K*_D_) determined by SPA (*y*-axis) and filtration (*x*-axis). For (partial) agonists LUF5834, CCPA, and NECA, only the low affinity state of the receptor was taken into account due to the presence of 1 mM GTP in the competition association experiments (**c**). Correlation of the negative logarithm of hA_1_R ligands’ affinity (p*K*_i_) from displacement studies of specific [^3^H]-DPCPX binding from hA_1_R membranes and the negative logarithm of hA_1_R ligands’ kinetic *K*_D_ (p*K*_D_) determined by SPA (**d**) and filtration (**e**). For (partial) agonists LUF5834, CCPA and NECA, only the low affinity state of the receptor was taken into account due to the presence of 1 mM GTP in the competition association experiments

The other unlabeled ligands included the hA_1_R partial agonist LUF5834 and full agonists CCPA and NECA. Their *k*_on_ and *k*_off_ values were determined in both SPA and filtration assays, in the presence of 1 mM GTP (Figs. 5, 6, and 7 and Table 3). The current mathematical model does not allow the calculation of two receptor states with corresponding kinetic parameters; the inclusion of GTP in the assay forces the receptor to be in one lower affinity, G protein-uncoupled state only. With this restriction, the kinetic parameters of both partial and full agonists were determined as conveniently as the two antagonists (Table 3). The kinetic profiles (*k*_on_ and *k*_off_) of all hA_1_R ligands obtained by SPA were in good agreement with the results from filtration (Fig. 8a, b). Due to its irreversible binding nature, FSCPX was not included in the correlation. The correlation between kinetic *K*_D_ values from either SPA or filtration assay was high too (Fig. 8c). Finally, with data from all experiments at hand, we concluded that the equilibrium *K*_i_ and kinetic *K*_D_ values from both SPA technology and filtration assay were also highly correlated (Fig. 8d, e).

## Discussion

In this study, we developed and validated a method based on the principles of a scintillation proximity assay (SPA) for the determination of kinetic characteristics of GPCR ligands. The adenosine A_1_ receptor was used as our workhorse, together with a number of reference ligands with divergent characteristics. In the following, we discuss the benefits and relatively minor concerns of the approach.

### Advantages of SPA technology

We substituted a standard filtration assay by SPA technology to determine the kinetics of the drug-receptor interaction. In that setting, the most obvious improvement is that the event of ligand association and dissociation to and from the receptor can be measured almost in real time without washing steps which are indispensable in a filtration assay. This improvement brings the benefit of great efficiency in kinetic radioligand binding experiments. For example, in the filtration format, association and dissociation kinetics of [^3^H]-DPCPX to and from hA_1_R are determined separately, because it is impractical to perform both association and dissociation experiments in one 96-well plate. However, in the SPA format, only a single well is required to record such a full curve (Fig. 2). This also brings impressively improved throughput in the competition association assay of unlabeled ligands with [^3^H]-DPCPX as the radioligand. In an individual filtration experiment, to measure competition association, a whole 96-well plate is used, allowing for two duplicate curves from three different concentrations of unlabeled ligand and a control curve. However, with SPA, only a few wells are required to obtain the same results, as is shown in Figs. 3, 4, 5, 6, and 7. This is due to the six-detector panel operating simultaneously in the counter, allowing a rich data collection of six wells simultaneously. On average, the duration of an *n* = 3 competition association experiment by SPA is reduced to 1–2 days from almost 1 week of practical work by filtration, with the additional benefit of much less bench time.

The SPA technology not only improved the speed and throughput of the kinetic radioligand binding experiments but also provided more, more precise and more accurate data. The competition association experiment is based on the Motulsky-Mahan model for competition association, which requires a substantial number of data points for plotting curves and subsequent analysis. In other words, the more data points obtained, the more accurate *k*_on_ and *k*_off_ can be determined. The SPA technology enables to acquire a great number (more) of data points, from one well rather than separate tubes (more precise). In this case of a 2-h experiment, one well on a 96-well plate was sampled every 30 s, yielding a total of 240 data points in one curve, while in the comparable filtration assay, there are only a labor-intensive dozen or so (Figs. 3, 4, 5, 6, and 7). A further reason of concern in the filtration assay is that the separation step only differentiates bound from unbound ligand, irrespective of whether the binding process itself consists of several steps [7] or that weaker interactions are broken such that only a fraction of receptor-ligand complexes might be detected after washing [30]. In the absence of filtration steps, SPA technology provides the possibility to collect this information without these caveats (more accurate). Lastly, overall financial expenses are favorable. Although the beads come at a price, the hugely reduced number of wells makes the experiment very cost-effective.

### Differences in SPA technology from filtration assay

SPA is a homogeneous bead-based technique, in which the receptor membrane protein is coupled with a certain type of SPA bead. Although there are several approaches to add SPA beads to the reaction (such as a precoupled format, a simultaneous addition (“T = 0”) format, or delayed addition format [10]), for kinetic experiments, precoupling of the cell membranes with SPA beads is necessary. The convenience of doing so is that bead and membrane are treated as a single reagent, thus reducing the time to dispense an assay, and there is no issue of membrane-bead diffusion. More importantly, the membrane-bead ratio needs to be optimized to generate a useful specific radioligand binding “window.” In our case, 5 μg of hA_1_R membrane protein was associated with 1 mg of WGA-PVT beads. Adding an excess of SPA beads would ensure that all the membranes are captured and a maximum signal is obtained, but the excess might equally contribute to an increased background signal [1]. It should be realized that besides normal non-specific binding (NSB), another background signal called non-proximity effect (NPE) may play a role. This was clearly observed in kinetic assays. The NPE is to describe that if a radioligand stays in close proximity, the bead would be activated irrespective of whether the radioligand is bound to the bead or membrane-bead mixture [2]. From Fig. 2a, it seems that [^3^H]-DPCPX did not fully dissociate from the hA_1_R as was the case in the filtration assay (Fig. 2b), with an elevated baseline of approx. 10 % of radioligand binding. This observation does not necessarily mean there was still 10 % of [^3^H]-DPCPX binding to the hA_1_R but rather indicates that some of the liberated [^3^H]-DPCPX stayed in the proximity of the bead, yielding a weak signal.

In the SPA technology, there is no need to add liquid scintillation fluid after harvesting as in filtration assays. As a consequence, the typical CPM-DPM conversion from the spectrometer’s counting efficiency as a result of quenching cannot be done [31–33]. As all events take place at the surface of the SPA bead [2], the light-emitting process cannot be quenched. A further different and unique aspect of SPA technology and corresponding equipment is that samples are monitored from both top and bottom and a count is only a count when top and bottom detectors both record the light quant within a certain amount of time. It is possible that a light quant is emitted at the top of the well by dispersion by the beads, while this quant is not seen by the bottom detector. The counter has the possibility to make a correction for this and therefore the CPM measured with SPA is referred to as CCPM which stands for “corrected” CPM (the *x*-axis of Figs. 5a, 6a, and 7a) [33].

### Challenges for SPA technology

Despite the many benefits of using SPA technology, there are also challenges in SPA radioligand binding studies. As mentioned above, the higher background signal of SPA consists of NSB and/or NPE. The NPE can be reduced by centrifuging the beads or allowing them to settle prior to counting, and by increasing the volume of the assay, but all these work-around solutions are either not feasible or impractical in kinetic radioligand binding experiments.

Secondly, although not much of an issue in our current experiments, the window of specific binding may need further consideration. Along this vein, one may try different SPA beads. There are two basic types of SPA beads: one is composed of plastic-based polyvinyltoluene (PVT), the other is silica-based yttrium silicate (Ysi). In general, PVT beads are bigger in size, in a regular ball shape, but lighter, while Ysi beads are crystal amorphous solids, which are heavier [2, 10]. Although in our case of the hA_1_R we chose the PVT beads, it was recently reported that two types of Ysi beads were used for radioligand binding studies on the adenosine A_2A_ receptor [34, 35]. Apart from the abovementioned beads designed for photomultiplier tube/well-based counters, there is another type of yttrium-based beads optimized for sensitive imaging-based detectors, the red-shifted yttrium oxide (YO) SPA bead. Both Ysi and YO SPA beads have been reported to enable HTS and to improve on the filtration method [36, 37].

Thirdly, even in a fast operation, the first 30 s of ligand association or dissociation cannot be recorded, as it takes time to have the spectrometer place the detectors above the wells and measure scintillations [7]. This is not an issue in our case of the hA_1_R, but it can be crucial with fastly associating ligands. This could be improved by using an automatic injection module inside the counter, although this is currently not provided. Lastly, the temperature inside the counting chamber is fixed and slightly higher than room temperature (in our experiments, the temperature was 28 ± 1 °C). In a typical filtration assay, kinetic radioligand binding experiments can be performed at lower or higher temperatures according to the characteristics of the radioligand—target interaction. Although in some scintillation counters it is possible to adjust temperature from 19 to 35 °C [34], a broader choice of assay temperatures would be highly advantageous.

## Conclusion

We reported a rapid and reliable technique, the scintillation proximity assay (SPA) technology, for kinetic radioligand binding studies on a prototypic GPCR, the human adenosine A_1_ receptor (hA_1_R). The SPA technology was of great benefit, as it monitored the event of radioligand binding in a single well in almost real time, which is impossible in traditional filtration assays. Even in the otherwise most laborious of all kinetic assays, the competition association assay, the kinetic profiles (*k*_on_ and *k*_off_) of unlabeled ligands for the hA_1_R were reliably and quickly determined and agreed very well with the same parameters in a filtration assay performed simultaneously. In conclusion, SPA is a very promising technique to determine the kinetic profiles of the drug-target interaction in the early phase of drug discovery. Its robustness and potential high-throughput may render this technology a preferred choice for further kinetic studies.
